# From spatial to social competence: The association between spatial ability and prosocial behaviour in childhood

**DOI:** 10.1111/bjdp.70001

**Published:** 2025-06-19

**Authors:** Dimitris I. Tsomokos, Eirini Flouri

**Affiliations:** ^1^ Department of Psychology and Human Development, Institute of Education University College London London UK

**Keywords:** child development, prosocial behaviour, prosociality, social cognition, spatial ability

## Abstract

This study investigated the nature of the association between spatial ability and prosocial behaviour in middle childhood. We used a general‐population longitudinal survey from the United Kingdom, which allowed us to control for a wide range of area, family and child covariates, including early verbal ability and parenting, in a large sample (*N* = 13,355, 51% male). The study's primary aim was to determine whether intrinsic‐dynamic spatial skills predicted prosocial behaviour and vice versa across ages 5 and 7 years. The results from cross‐lagged panel models with various levels of adjustment indicated that both paths were significant and equally strong. However, when also controlling for verbal ability and parenting practices, verbal ability (but not parenting) confounded the path from prosocial behaviour at age 5 to spatial ability at age 7. Therefore, only the path from spatial to social skills remained significant after adjustment for all confounders. Sex‐stratified analyses did not reveal significant differences between the paths for males and females. The present study contributes to our understanding of social and cognitive development in children, highlighting the impact of spatial skills across the social domain. The findings have implications for educational curricula in the early years and primary school.

**What is already known on this subject?**

- Experimental research has established a causal link between visual perspective‐taking (an important spatial skill) and theory of mind (a key social cognitive skill).
- However, it is not known whether the relationship between spatial ability and social competence more broadly is reciprocal.

**What does the present study add?**

- Using data from a large, nationally representative UK birth cohort, we investigated the bidirectional association between spatial ability and prosocial behaviour at ages 5 and 7, controlling for demographic, socioeconomic, and a range of confounders that included parenting, parental mental health, and the child’s verbal ability.
- We found that spatial ability at age 5 was significantly associated with prosocial behaviour at age 7 even after full adjustment, but the association between prosocial behaviour at age 5 and spatial ability at age 7 was confounded by early verbal ability.

## INTRODUCTION

Acts of kindness, spontaneous sharing and empathy, which are manifestations of prosocial behaviour, enhance effective social interactions and promote social competence (Grusec et al., 2002). In childhood, these fundamental social skills support social and psychological adjustment (Memmott‐Elison et al., 2020; Spataro et al., 2020), and are linked with numerous positive outcomes throughout the lifespan. These include self‐esteem (Coulombe & Yates, 2022; Preston & Rew, 2022), life satisfaction, happiness (Dunn et al., 2008) and subjective well‐being (Hui et al., 2020). In this study, we explore the question of whether prosocial behaviour in children is also linked to spatial ability, a skill many would consider unrelated to it.

Spatial ability is a cognitive skill (Bjorklund, 2022; Cimadevilla & Piccardi, 2020) that involves understanding, recalling and mentally manipulating spatial information (Alkouri, 2022; Kyllonen & Gluck, 2003). It develops with age during childhood, before stabilizing in adolescence (Farrell Pagulayan et al., 2006), and is strongly linked to academic performance in science and mathematics (Gilligan et al., 2017; Hawes et al., 2022). Here, we argue that a link with prosocial behaviour is plausible via social cognition and broader social competence. Social cognition is a fundamental requirement for prosocial behaviour (Conte et al., 2018; Denham, 1986) that also shares an element of self‐projection (Buckner & Carroll, 2007), in turn requiring spatial skills. Self‐projection takes many forms, from remembering the past (episodic memory) and thinking about the future (prospection) to envisioning oneself in different places (arguably requiring spatial navigation) and taking the perspective of others (arguably requiring visual perspective taking). But they all rely on the ability to transcend the present moment and project oneself into different perspectives or scenarios (Jarvis & Miller, 2017; Soares et al., 2023; Spreng et al., 2009).

Experimental research has indeed linked *causally* spatial ability in children with a crucial social cognitive skill, theory of mind, via visual perspective taking (Tian et al., 2021). Theory of mind is the ability to draw inferences about the mental states of others (Baron‐Cohen et al., 1985; Frith & Frith, 2007; Premack & Woodruff, 1978), therefore allowing one to anticipate others' behaviour (Sebanz et al., 2006), and communicate more efficiently (de Villiers, 2021; Dunbar, 1998)—all of which are crucial skills for the development of children's social competence and therefore prosocial behaviour (Huber et al., 2019). Related work with autistic individuals has also shown that impairments in spatial navigation or wayfinding are associated with social cognition deficits (Lind et al., 2013; Noel et al., 2020; Presley, 2021; Smith, 2015; Yang et al., 2021), supporting a grounded cognition view (Barsalou, 2008; Barsalou et al., 2003; Niedenthal et al., 2005) that visuospatial perspective taking is the grounding of psychological perspective taking (Erle & Topolinski, 2017; Hamilton et al., 2009; McGarry et al., 2021; Pearson et al., 2013). Grounded cognition posits that cognitive processes are grounded in sensorimotor systems and involve embodied simulations—for instance, the feeling of affective warmth and relational closeness may involve an embodied simulation of physical proximity. Previous research has experimentally induced a series of embodied simulations in various tasks and showed that psychological perspective taking was causally influenced by visual‐spatial perspective taking—providing evidence of the grounded nature of this social skill (Erle & Topolinski, 2015, 2017). In addition, experiments on so‐called ‘egocentric transformation’ (Jola & Mast, 2005; Surtees et al., 2013) have shown that the disparity in the orientation between a participant and an agent influences perspective taking (Frith & De Vignemont, 2005; Proulx et al., 2016; Xie et al., 2018).

Arguably, however, the link may also run in the opposite direction, such that prosocial skills could predict spatial skills, since prosocial interactions provide a context for developing a range of cognitive abilities in children. It has recently been shown, for instance, that children engaging in prosocial behaviours show improvements in language and social cognitive skills that include better understanding others' perspectives (Tsomokos & Raviv, 2024). Prosocial interactions with peers during play may be a special case for such a context in children. Children frequently engage in play scenarios that require them to think about where others are looking or how they view an object or situation. Such experiences may strengthen their capacity to mentally manipulate objects and understand spatial relationships, thereby contributing to enhanced spatial skills over time. For instance, when children play, they must often understand and anticipate the positions and intentions of their peers, which may enhance their spatial awareness and dynamic spatial reasoning.

### The present study

Taken together, these findings suggest that spatial skills and prosocial behaviour in children may be reciprocally related. Such a link would also be supported by much evidence that social competence more broadly is associated with visual perspective taking and visual‐spatial skills in both adults (Shelton et al., 2012; Wolgast et al., 2020) and children (Sobel & Blankenship, 2021; Tanaś & Szarek, 2021; Tsoi & McAuliffe, 2020). We carried out this study to test this hypothesis, namely, that the link between spatial skills and prosocial behaviour in the general child population would be bidirectional. Our study used data from a large, longitudinal, general‐population birth cohort from the United Kingdom, the Millennium Cohort Study, which measured both spatial cognition (intrinsic‐dynamic skills) and prosocial behaviour in middle childhood, a critical developmental window for the establishment of both skillsets. Intrinsic spatial skills involve understanding the configuration of elements within an object (or a set of objects), such as how pieces of a puzzle fit together and dynamic spatial skills involve understanding and predicting changes in spatial configurations over time (e.g. predicting the movement of objects in space or the constantly updating of relative positions of objects in a changing environment). To ensure that any associations observed were not due to confounding, we controlled for several potential shared causes, including sex and ethnicity (Devine & Hughes, 2018; Lillard, 1998; Madison, 2021), family income and education (Ebert et al., 2017; Kotsopoulos et al., 2021; Levine et al., 2005), parenting and parental mental health (Assel et al., 2003; Licata et al., 2016; Pears & Moses, 2003), and early language ability (de Villiers, 2021; Miller, 2006; Mizzi, 2017).

## METHODS

### Participants and analytic sample

The Millennium Cohort Study (MCS) uses regular survey sweeps that track the lives of around 19,000 children born in England, Scotland, Wales and Northern Ireland during 2000–2002 (Joshi & Fitzsimons, 2016). The initial sampling frame for this survey (Plewis et al., 2004) was designed, so that families living in high child‐poverty areas were represented more than others, as were families living in areas with at least 30% ethnic minority populations in England. The MCS waves took place at age 9 months and ages 3, 5, 7, 11, 14, 17 and 23 years. UK Multi‐Centre Ethics Committees coordinated by the National Health Service in the four countries provided ethical approvals in each survey wave (Shepherd & Gilbert, 2019). Parents provided informed consent prior to any interviews during the childhood and adolescent waves. The children themselves provided their assent at the age 11 wave and their informed consent from the age 14 wave onwards.

At age 5 and 7, both prosocial behaviour and spatial ability were measured; therefore, the starting point of the present study was the age 5 wave (with all the confounders being taken from the previous wave at age 3). At the starting point, there were 15,575 cohort members who were singletons or first‐born twins/triplets. We required that these children—some of whom did not participate in the next wave at age 7 years—had complete data on both prosocial behaviour and spatial ability at baseline (age 5). Given this condition, 13,355 participants (51% male, 46% in disadvantaged neighbourhoods, 14% non‐White) remained in the analytic sample.

### Measures and procedures

#### Spatial ability (5 and 7 years)

This was assessed on both occasions using the same pattern construction task (British Ability Scales Second Edition, BAS II) (Hill, 2005). As explained elsewhere (Flouri & Tsomokos, 2024), the task consists of challenges where children need to replicate patterns using a set of blocks that vary in surface design, featuring either solid colours (yellow or black) or a dual‐tone (half‐yellow, half‐black) scheme. Participants are required to manipulate the blocks through rotation and arrangement to accurately match the given stimulus pattern. As such, the task measures intrinsic‐dynamic spatial skills (Uttal et al., 2013). Performance is evaluated based on the precision of block placement and orientation, as well as the speed of response. In our study, the scaled, age‐adjusted variable of performance ranged from 1 to 61, with higher scores indicating better spatial skills.

#### Prosocial behaviour (5 and 7 years)

This was measured with the prosocial behaviour scale of the Strengths and Difficulties Questionnaire (SDQ) (Goodman, 1997), ranging from 0 to 10. The scale is composed of 5 items, completed by the child's primary caregiver (predominantly the mother), on a Likert scale (0 for ‘not true’, 1 for ‘somewhat true’ and 2 for ‘certainly true’). The items asked the parent to indicate the degree to which the child is considerate of others' feelings; shares readily with others; is helpful when someone is hurt, upset or ill; is kind to younger children; and volunteers to help others (Cronbach's $\alpha_{5}=0.64$ and $\alpha_{7}=0.67$ at the age 5 and 7 waves, respectively).

#### Covariates at age 3 years

We controlled for the following covariates (all measured prior to the starting point, i.e. at the age 3 wave) that are known to correlate with both prosocial behaviour and spatial cognitive abilities in middle childhood. *Sex* was male or female at birth, as reported by the main respondent. *Ethnicity* was based on the UK Census‐defined categories (White, Indian, Pakistani and Bangladeshi, Black or Black British, Mixed, Other Ethnic group including Chinese or Other). *Income* was the household's OECD equivalized income (given in quintiles). *Maternal education* was the mother's highest educational level attained, based on the UK's National Vocational Qualifications classification and its equivalents (on a scale ranging from 1 to 6). *Maternal psychological distress* was provided by the self‐reported Kessler 6‐item scale, a numerical variable ranging from 1 to 25 (with higher scores indicating higher levels of distress) (Kessler et al., 2010). *Parenting (mother's low responsivity)* was measured with the Home Observation Measurement of the Environment‐Short Form (HOME‐SF) by Caldwell and Bradley (1984) when the interviewer visited the child's home and observed interactions of the mother–child dyad. The score is a numerical variable from 0 to 5, where 0 indicates that all of the following positive interactions were observed during the visit: the mother (1) conveyed positive feeling in her voice; (2) conversed with the child at least twice; (3) answered the child's questions or requests verbally; (4) spontaneously praised the child's behaviour or qualities twice; (5) caressed, kissed or cuddled the child. Therefore, higher scale scores indicate fewer positive interactions. *Verbal ability* was measured with the BAS II Naming Vocabulary assessing expressive language skills (we used the age‐standardized T‐score, a numerical variable ranging from 1 to 63). Further information on all the variables in the present study can be found in the additional Supplemental Online Material (SOM, 2024) published permanently on the Open Science Framework website.

### Analytic strategy

#### Sample bias, missing data and pairwise correlations

We started with a descriptive analysis, performed for two reasons: first, to test for any systematic differences between participants excluded from the analytic sample and those included in it; second, to identify patterns of missingness in the analytic sample. In this preliminary analysis, we also calculated pairwise correlations between the numerical variables in the analytic sample.

#### Survey‐weighted, imputed cross‐lagged panel models (CLPMs)

To answer our research question, we fitted CLPMs with survey‐weighted, imputed data. Missing data were imputed using multiple imputation by chained equations (Raghunathan et al., 2001), and estimates and standard errors across imputed data sets were combined with Rubin's rules (Rubin, 1987). Calculations were performed in R (R Core Team, 2021) with the ‘mice’ (van Buuren & Groothuis‐Oudshoorn, 2011) and ‘lavaan’ packages (Rosseel, 2012). We used m=25 imputation runs in each case—details of this process and the full code output are included in the SOM (2024). We ran three models. Model 1 controlled only for sex. Model 2 additionally controlled for stratum (i.e. the area ‘type’ across UK countries that the MCS sample design was built on), ethnicity, household income, maternal education and maternal psychological distress. In Model 3, we further controlled for verbal ability and parenting. Finally, in an additional analysis stratified by the child's sex, the third model was refitted separately in males and females in view of the evidence for sex differences in both spatial cognition and prosocial behaviour.

## RESULTS

### Sample bias and missing data

Those excluded from the analytic sample (14% of the total eligible sample at the age 5 wave) were more likely to come from ethnically diverse areas in England (27%), be of non‐White backgrounds (33%) and from lower total household incomes ($d=-0.24,95\%\mathrm{CI}\;\left[-0.28,-0.19\right]$). Demographic information and other variables' details are provided in Table 1. The analytic sample had complete data on stratum, sex, verbal ability (as well as prosocial behaviour and spatial ability at age 5, by definition). The number of missing values for the remaining variables ranged from just 72 for ethnicity (<1%) and 458 for maternal education (<3.5%) to 1463 for maternal psychological distress (11%). The maximum missingness (13%) occurred for the two key variables at age 7 years, namely, prosocial behaviour and spatial ability. Values were not Missing Completely at Random (MCAR), as confirmed by Little's MCAR test (Little, 1988) as well as a between‐group test for records with missing values (n=6185) and the complete records group (n=9390) (SOM, 2024).

**TABLE 1:** Sample bias analysis: Unweighted variable distribution between the analytic sample and the rest of the MCS at age 5 years.

Characteristic	Rest of sample *N* = 2220	Analytic sample *N* = 13,355	*p*‐Value^a^
Sex, *n* (%)			.95
Male	1134 (51)	6813 (51)	
Female	1086 (49)	6542 (49)	
Area stratum, *n* (%)			**<.001**
England – Advantaged	363 (16)	3843 (29)	
England – Disadvantaged	578 (26)	3297 (25)	
England – Ethnic	592 (27)	1367 (10)	
Wales – Advantaged	77 (3.5)	615 (4.6)	
Wales – Disadvantaged	208 (9.4)	1359 (10)	
Scotland – Advantaged	99 (4.5)	833 (6.2)	
Scotland – Disadvantaged	132 (5.9)	748 (5.6)	
N. Ireland^b^ – Advantaged	57 (2.6)	528 (4.0)	
N. Ireland^b^ – Disadvantaged	114 (5.1)	765 (5.7)	
Ethnicity, *n* (%)			**<.001**
White	1455 (66)	11,429 (86)	
Mixed	85 (3.9)	363 (2.7)	
Indian	83 (3.8)	318 (2.4)	
Pakistani and Bangladeshi	356 (16)	659 (5.0)	
Black or Black British	148 (6.7)	361 (2.7)	
Other ethnic group	66 (3.0)	153 (1.2)	
(Missing)	27	72	
Income, mean (SD)	2.61 (1.38)	2.94 (1.40)	**<.001**
(Missing)	72	115	
Maternal education, mean (SD)	2.91 (1.54)	3.59 (1.41)	**<.001**
(Missing)	161	458	
Verbal ability, mean (SD)	27 (15)	33 (13)	**<.001**
Maternal psychological distress, mean (SD)	4.6 (4.2)	4.2 (3.7)	.**018**
(Missing)	601	1463	
Mother's low responsivity, mean (SD)	0.38 (0.88)	0.23 (0.69)	**<.001**
(Missing)	234	970	
Prosocial behaviour (age 5), *n* (%)	8.15 (1.46)	8.38 (1.67)	.24
(Missing)	2194	0	
Prosocial behaviour (age 7), *n* (%)	8.13 (2.08)	8.60 (1.62)	.**002**
(Missing)	1981	1708	
Spatial ability (age 5), mean (SD)	24 (12)	31 (11)	**<.001**
(Missing)	1813	0	
Spatial ability (age 7), mean (SD)	29 (12)	34 (11)	**<.001**
(Missing)	1903	1750	

*Note*: Bold, *p* < .05.

^a^ Pearson's Chi‐squared test; Wilcoxon rank‐sum test.

^b^ N. = Northern.

### Correlations

Pairwise correlations between all the numerical variables in the analytic sample are presented in Table 2. The strongest correlation among the covariates was between income and maternal education (r=.53,p<.001).

**TABLE 2:** Correlations between numerical variables (pairwise complete observations).

	(1)	(2)	(3)	(4)	(5)	(6)	(7)	(8)
Income (1)								
Maternal education (2)	.**53**							
Verbal ability (3)	.**30**	.**28**						
Spatial ability at age 7 years (4)	.**23**	.**22**	.**26**					
Spatial ability at age 5 years (5)	.**19**	.**19**	.**28**	.**54**				
Prosocial behaviour at age 5 years (6)	.**08**	.**08**	.**13**	.**07**	.**11**			
Prosocial behaviour at age 7 years (7)	.**09**	.**07**	.**13**	.**06**	.**10**	.**51**		
Maternal psychological distress (8)	**−.21**	**−.12**	**−.10**	**−.09**	**−.07**	**−.10**	**−.10**	
Mother's low responsivity (9)	**−.15**	**−.14**	**−.14**	**−.09**	**−.09**	**−.06**	**−.05**	.**08**

*Note*: Pearson's correlation coefficients (bold for *p* < .05).

### Survey‐weighted, imputed cross‐lagged panel models (CLPMs)

The cross‐lagged paths were significant with small to medium effect sizes in all three models—for effect size classification in CLPM paths, we consulted Orth et al. (2022). In the minimally adjusted case (Model 1), where we only controlled for the cohort member's sex, spatial ability at age 5 years was positively associated with prosocial behaviour at age 7 ($b_{S5\to P7}=0.006,\mathrm{SE}=0.001,z=3.978,p<.001,95\%\mathrm{CI}\;\left[0.003,0.009\right]$, standardized coefficient $\beta_{S5\to P7}=0.038$) and, conversely, prosocial behaviour at age 5 years was associated with spatial ability at age 7 ($b_{P5\to S7}=0.219,\mathrm{SE}=0.065,z=3.395,p=.001,95\%\mathrm{CI}\;\left[0.093,0.346\right]$, $\beta_{P5\to S7}=0.033$).

**FIGURE 1:**
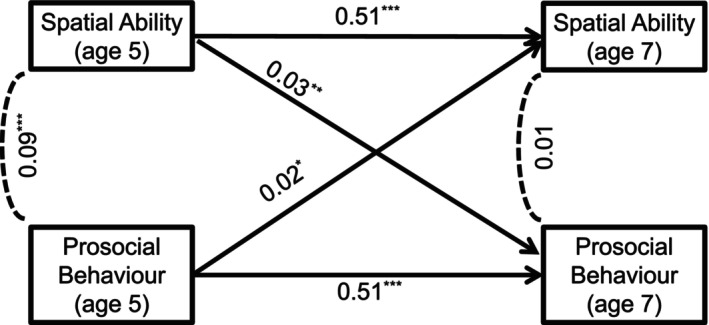
Cross‐lagged panel model for spatial ability and prosocial behaviour (ages 5 and 7) in the adjusted case (Model 2), without controlling for parenting and verbal ability: Covariances and standardized estimates (for the autoregressive and cross‐lagged paths) (N=13,355, imputed, survey‐weighted). *p < .05, **p < .01, ***p < .001.

In Model 2, where we also adjust for stratum, ethnicity, income, maternal education and maternal distress, these bidirectional associations remained robust, with spatial ability predicting prosocial behaviour ($\beta_{S5\to P7}=0.030$) and prosocial behaviour predicting spatial ability ($\beta_{P5\to S7}=0.022$). Results for these two cases and the fully adjusted model can be found in Table 3, and complete information on these models (including standardized coefficients and exact *p*‐values) can be found in the Supplemental Online Material, ‘Full Results’ section (SOM, 2024). The standardized estimates for Model 2 are depicted in the diagram of Figure 1.

**TABLE 3:** Cross‐lagged paths for prosocial behaviour and spatial ability at 5 and 7 years: Controlling for sex only (Model 1); adjusted model (2); additionally controlling for verbal ability and parenting (3).

	Model 1	Model 2	Model 3
Prosocial behaviour, 7 years			
Spatial ability, 5 years	0.01 (0.00)***	0.00 (0.00)**	0.00 (0.00)*
Prosocial behaviour, 5 years	0.50 (0.01)***	0.50 (0.01)***	0.49 (0.01)***
Spatial ability, 7 years			
Spatial ability, 5 years	0.57 (0.01)***	0.53 (0.01)***	0.52 (0.01)***
Prosocial behaviour, 5 years	0.22 (0.06)***	0.15 (0.06)*	0.10 (0.06)

*Note*: *p < .05, **p < .01, ***p < .001 | N=13,355 | Survey‐weighted, imputed models showing unstandardized estimates (std. errors). See Table S1 for full details. As these are all saturated models, the fit indices are omitted.

In Model 3, which further controlled for parenting and verbal ability, only the path from spatial ability at age 5 to prosocial behaviour at age 7 remained significant ($b_{S5\to P7}=0.003,\mathrm{SE}=0.002,z=2.086,p=.037,95\%\mathrm{CI}\;\left[0.000,0.006\right]$, standardized $\beta_{S5\to P7}=0.021$, $95\%\mathrm{CI}\;\left[0.001,0.041\right]$). To test whether this path was stronger, we conducted a formal test of equality between the standardized $\beta_{S5\to P7}$ and $\beta_{P5\to S7}$ (Klopp, 2020) and found that the two paths were equally strong (Wald test statistic W=0.842,p=.359). Details are included in Table 3 (see Appendix S1, Table S1, for full information). Notably, as can be seen in the full Table (S1), only the cohort member's verbal ability at age 3 (not the parenting they received at that age) confounds the cross‐lagged path between prosocial behaviour at age 5 and spatial ability at age 7.

Finally, in a sex‐stratified model (imputed, fully adjusted Model 3 refitted separately for males and females), we found no evidence of sex‐based differences in the cross‐lagged paths. In particular, a formal test (Paternoster et al., 1998) of the two coefficients, $\beta_{S5\to P7}^{m,f}$ and $\beta_{P5\to S7}^{m,f}$, did not reveal any statistically significant difference between males (N=6813) and females (N=6542). Complete information on these models can be found in the last section of the SOM (2024).

## DISCUSSION

The results of the present study did not support our hypothesis that prosocial behaviour and spatial ability are associated bidirectionally in middle childhood (at ages 5 and 7 years) in the general population. In our UK sample, spatial ability at age 5 years was significantly associated with prosocial behaviour at age 7, even after adjustment for prosocial behaviour at baseline (age 5), as well as demographic and socioeconomic confounders, maternal psychological distress, parenting and verbal ability. However, while prosocial behaviour at age 5 was significantly associated with spatial ability at age 7 even after controlling for spatial ability at age 5, demographic and socioeconomic confounders, parenting and maternal psychological distress, it was not linked to later spatial ability after adjusting for verbal ability as well. In either case, the strength of the two cross‐lagged paths was found to be equal. In a sex‐stratified analysis, we found that they were also equally strong between the male and female groups.

To the best of our knowledge, this is the first investigation of whether there is a bidirectional link between spatial ability and prosocial behaviour in the general child population. The observed associations would need to be experimentally tested to establish causality and replicated across different environments to examine how they generalize to other contexts. Assuming they remain robust, then the fact that spatial abilities can support the development of prosocial skills implies that spatial cognitive training in early years could translate to social competencies later in childhood. According to our findings, however, strengthening prosocial skills (e.g. helping and sharing behaviours) would not significantly improve intrinsic‐dynamic spatial skills over and above the strengthening of verbal ability. Overall, then, the present findings support the case for the inclusion (and greater prominence) of spatial skills training in early childhood and primary school educational curricula (Pritulsky et al., 2020) as benefits likely generalize across child development domains. On the contrary, the promotion of prosocial behaviour could strengthen cognitive, and particularly linguistic, skills (Tsomokos & Raviv, 2024), but, according to our findings, not spatial ability. We acknowledge that an important open problem is to further delineate the contribution of executive function skills (working memory, and particularly spatial working memory, inhibitory control, sustained attention and cognitive flexibility) in the link between prosocial behaviour and spatial ability (Hawes et al., 2019; O'Toole et al., 2017; Traverso et al., 2015, 2020; Yazdani et al., 2021).

We must also acknowledge several study limitations. First, the observational nature of the survey precludes any definitive conclusions about causality. Second, the use of a standardized assessment for spatial ability and a well‐established scale for prosocial behaviour, while rigorous, may not capture the full complexity and nuance of these constructs. Third, the study's focus on the UK may limit the generalisability of our findings to other cultural, social and educational contexts. Fourth, on a more methodological level, the lack of more than two repeat assessments of both spatial ability and prosocial behaviour in MCS does not allow us to draw any conclusions in relation to within‐person variations in our two constructs over time (Hamaker et al., 2015). Finally, the effect sizes were small, but not trivially so, based on effect classifications for cross‐lagged panel models (Orth et al., 2022) and recent considerations regarding the impact of small effects at a population level (Carey et al., 2023).

Despite these limitations, the observed associations between the social and cognitive skills we examined underscore the interconnectedness of prosocial behaviour and both verbal and spatial abilities. This aligns with evidence supporting self‐projection (Buckner & Carroll, 2007; Schacter et al., 2012; Smallwood et al., 2021; Yeshurun et al., 2021), which suggests that humans have the cognitive ability to transcend their immediate sensory experience and take different temporal, spatial or social cognitive perspectives. This allows us to remember (i.e. project into the past), imagine (project into the future), find our way or navigate different places (project into space) and consider/understand or empathize with other people's thoughts and feelings (project into others' psychological perspectives).

‘Grounding’ social skills on spatial abilities does not stand in opposition to the view supporting the key role of mental representation in social development (Dweck & London, 2004). Arguably, for children in our age group, grounded representations are more accessible compared with more developed, abstract representations. In the context of spatial skills, in particular, grounded representations involve physically simulating or imagining spatial positions and movements, which aligns well with the way young children understand and interact with the world (Hawes et al., 2022; Lee‐Cultura & Giannakos, 2020). This grounded approach may facilitate the embodied experience of ‘seeing’ from another's point of view, which supports social cognition and social competence. By contrast, abstract representations require a higher level of cognitive abstraction, which may not be as readily available in middle childhood.

On a broader level, our findings echo the basic tenets of *topological psychology* (Lewin, 1936), which has been largely ignored due to its reliance on insufficiently developed concepts from the field of mathematical topology (Back, 1992). However, Lewin's ideas have been revisited in recent years, and the potential use of topological concepts in psychology is beginning to be appreciated (Skowron & Wójtowicz, 2021). Lewin's concept of the *lifespace* is of particular relevance in our context, as it encompasses the totality of a person's mental reality at any given moment, including perceptual and motivational states. This includes physical space, and its objects as perceived by the individual, as well as the individual's needs, desires, affective states and social relationships. In Lewin's topological formulation, a child's perception of his or her psychosocial space—crucially including social relationships—and the child's perception of the physical‐material environment are both regions of the lifespace, and there is a homeomorphism between them. Therefore, the social gains of spatial skills reported here may be suggestive of a latent competence in navigating two different psychological regions.

## AUTHOR CONTRIBUTIONS

**Dimitris I. Tsomokos:** Conceptualization; investigation; writing – original draft; methodology; visualization; writing – review and editing; formal analysis; software; project administration; data curation; resources; funding acquisition. **Eirini Flouri:** Conceptualization; methodology; writing – review and editing; project administration; data curation; supervision; investigation.

## FUNDING INFORMATION

D.I.T. was partially supported by Alphablocks Nursery School Ltd.

## CONFLICT OF INTEREST STATEMENT

The authors report no biomedical financial interests or other potential conflicts of interest.

## Supporting information


Appendix S1

## Data Availability

The data necessary to reproduce the analyses presented here are publicly accessible. Data are available under licence from the UK Data Service and Jisc (see, for instance, https://ukdataservice.ac.uk/). This dataset was used for secondary data analysis; it had been fully anonymized by the UK Data Service. The output of the code to reproduce the analyses (in the form of R markdown) is publicly available on the OSF website as referenced in the manuscript.
