# Supplementary material for: From spatial to social competence: The association between spatial ability and prosocial behaviour in childhood
Source: Br J Dev Psychol. 2025 Jun 19;44(1):20–32. doi: 10.1111/bjdp.70001 (PMC12884366; doi:10.1111/bjdp.70001)
Supplement: Supplementary file 1 — Appendix S1 [file BJDP-44-20-s001.docx]

**Appendix**

**Table S1.** Three cross-lagged panel models for prosocial behaviour and spatial ability (5 and 7 years): Unadjusted (Model 1); Moderately adjusted (2); Fully adjusted (3).

|  | Model 1 | Model 2 | Model 3 |
| --- | --- | --- | --- |
|  | Regression Slopes – unstandardised est. (std. err.) | | |
| **Prosocial behaviour, 7y** |  |  |  |
| Spatial ability, 5y | 0.01(0.00)^***^ | 0.00(0.00)^**^ | 0.00(0.00)^*^ |
| Prosocial behaviour, 5y | 0.50(0.01)^***^ | 0.50(0.01)^***^ | 0.49(0.01)^***^ |
| Sex: Female | 0.30(0.03)^***^ | 0.30(0.03)^***^ | 0.29(0.03)^***^ |
| England - Disadvantaged |  | 0.04(0.04) | 0.04(0.04) |
| England - Ethnic |  | -0.01(0.08) | 0.01(0.08) |
| Northern Ireland - Advantaged |  | -0.03(0.07) | -0.03(0.07) |
| Northern Ireland - Disadvantaged |  | 0.01(0.07) | 0.02(0.07) |
| Scotland - Advantaged |  | -0.02(0.05) | -0.03(0.05) |
| Scotland - Disadvantaged |  | 0.05(0.05) | 0.04(0.05) |
| Wales - Advantaged |  | 0.20(0.07)^**^ | 0.20(0.07)^**^ |
| Wales - Disadvantaged |  | 0.04(0.06) | 0.04(0.06) |
| Black/Black British |  | 0.21(0.12) | 0.24(0.12) |
| Indian |  | -0.02(0.11) | 0.02(0.11) |
| Mixed |  | -0.17(0.09) | -0.17(0.09) |
| Other ethnic group |  | 0.01(0.17) | 0.06(0.17) |
| Pakistani & Bangladeshi |  | 0.17(0.08)^*^ | 0.22(0.08)^**^ |
| Income |  | 0.05(0.01)^***^ | 0.05(0.01)^***^ |
| Maternal education |  | 0.00(0.01) | -0.01(0.01) |
| Maternal psychological distress |  | -0.01(0.00)^***^ | -0.01(0.00)^***^ |
| Mother’s low responsivity |  |  | -0.03(0.02) |
| Verbal ability |  |  | 0.01(0.00)^***^ |
| **Spatial ability, 7y** |  |  |  |
| Spatial ability, 5y | 0.57(0.01)^***^ | 0.53(0.01)^***^ | 0.52(0.01)^***^ |
| Prosocial behaviour, 5y | 0.22(0.06)^***^ | 0.15(0.06)^*^ | 0.10(0.06) |
| Sex: Female | -0.46(0.19)^*^ | -0.35(0.20) | -0.52(0.20)^**^ |
| England - Disadvantaged |  | -0.72(0.28)^**^ | -0.68(0.28)^*^ |
| England - Ethnic |  | -0.85(1.04) | -0.54(1.05) |
| Northern Ireland - Advantaged |  | 0.85(0.61) | 0.87(0.61) |
| Northern Ireland - Disadvantaged |  | 0.34(0.60) | 0.37(0.60) |
| Scotland - Advantaged |  | 1.41(0.56)^*^ | 1.27(0.56)^*^ |
| Scotland - Disadvantaged |  | -0.19(0.44) | -0.27(0.44) |
| Wales - Advantaged |  | 0.65(0.72) | 0.69(0.72) |
| Wales - Disadvantaged |  | 0.55(0.36) | 0.61(0.36) |
| Black/Black British |  | -3.42(1.37)^*^ | -3.00(1.39)^*^ |
| Indian |  | -1.12(0.75) | -0.61(0.76) |
| Mixed |  | -1.04(0.66) | -0.93(0.66) |
| Other ethnic group |  | 0.56(0.75) | 1.39(0.77) |
| Pakistani & Bangladeshi |  | -1.43(0.84) | -0.64(0.85) |
| Income |  | 0.49(0.08)^***^ | 0.40(0.09)^***^ |
| Maternal education |  | 0.56(0.08)^***^ | 0.46(0.08)^***^ |
| Maternal psychological distress |  | -0.08(0.03)^**^ | -0.07(0.03)^**^ |
| Mother’s low responsivity |  |  | -0.21(0.15) |
| Verbal ability |  |  | 0.07(0.01)^***^ |
| **Prosocial behaviour, 5y** |  |  |  |
| Sex: Female | 0.51(0.03)^***^ | 0.51(0.03)^***^ | 0.46(0.03)^***^ |
| England - Disadvantaged |  | -0.08(0.04)^*^ | -0.07(0.04) |
| England - Ethnic |  | 0.00(0.09) | 0.07(0.09) |
| Northern Ireland - Advantaged |  | -0.04(0.07) | -0.04(0.07) |
| Northern Ireland - Disadvantaged |  | -0.11(0.08) | -0.10(0.08) |
| Scotland - Advantaged |  | -0.08(0.05) | -0.10(0.05) |
| Scotland - Disadvantaged |  | -0.13(0.08) | -0.13(0.08) |
| Wales - Advantaged |  | 0.07(0.08) | 0.08(0.08) |
| Wales - Disadvantaged |  | 0.06(0.07) | 0.08(0.07) |
| Black/Black British |  | 0.05(0.15) | 0.14(0.16) |
| Indian |  | 0.23(0.13) | 0.33(0.13)^**^ |
| Mixed |  | -0.04(0.11) | -0.01(0.11) |
| Other ethnic group |  | -0.08(0.21) | 0.07(0.21) |
| Pakistani & Bangladeshi |  | -0.22(0.13) | -0.07(0.13) |
| Income |  | 0.02(0.01) | -0.00(0.01) |
| Maternal education |  | 0.06(0.02)^***^ | 0.04(0.02)^*^ |
| Maternal psychological distress |  | -0.04(0.00)^***^ | -0.04(0.00)^***^ |
| Mother’s low responsivity |  |  | -0.08(0.03)^*^ |
| Verbal ability |  |  | 0.01(0.00)^***^ |
| **Spatial ability, 5y** |  |  |  |
| Sex: Female | 1.93(0.22)^***^ | 1.93(0.22)^***^ | 1.36(0.22)^***^ |
| England - Disadvantaged |  | -1.10(0.47)^*^ | -0.94(0.47)^*^ |
| England - Ethnic |  | -0.52(0.85) | 0.30(0.86) |
| Northern Ireland - Advantaged |  | 0.69(0.72) | 0.71(0.72) |
| Northern Ireland - Disadvantaged |  | 0.10(0.85) | 0.20(0.85) |
| Scotland - Advantaged |  | -1.87(1.12) | -2.13(1.12) |
| Scotland - Disadvantaged |  | -2.76(0.58)^***^ | -2.84(0.58)^***^ |
| Wales - Advantaged |  | -0.26(0.74) | -0.15(0.74) |
| Wales - Disadvantaged |  | -0.76(0.58) | -0.59(0.58) |
| Black/Black British |  | -3.58(1.62)^*^ | -2.37(1.61) |
| Indian |  | -0.83(0.77) | 0.46(0.78) |
| Mixed |  | -1.05(0.75) | -0.73(0.75) |
| Other ethnic group |  | -0.08(0.99) | 2.03(0.97)^*^ |
| Pakistani & Bangladeshi |  | -2.47(1.11)^*^ | -0.33(1.12) |
| Income |  | 0.76(0.09)^***^ | 0.50(0.09)^***^ |
| Maternal education |  | 1.01(0.09)^***^ | 0.72(0.09)^***^ |
| Maternal psychological distress |  | -0.06(0.03)^*^ | -0.04(0.03) |
| Mother’s low responsivity |  |  | -0.47(0.24) |
| Verbal ability |  |  | 0.19(0.01)^***^ |
|  | Covariances | | |
| Prosocial behaviour, 7y ~ Spatial ability, 7y | 0.20(0.13) | 0.11(0.17) | 0.57(0.14) |
| Prosocial behaviour, 5y ~ Spatial ability, 5y | 1.89(0.24)^***^ | 1.47(0.23)^***^ | 1.10(0.22)^**^ |
| ^*^p<0.05, ^**^p<0.01, ^***^p<0.001 \| $N=13,355$ \| Survey-weighted, imputed cases. | | | |
